# Pro-social cognition: helping, practical reasons, and ‘theory of mind’

**DOI:** 10.1016/j.cognition.2015.08.006

**Published:** 2015-08-29

**Authors:** Johannes Roessler, Josef Perner

**Affiliations:** 1 Department of Philosophy, University of Warwick, Coventry, UK; 2 Centre for Cognitive Neuroscience and Department of Psychology, University of Salzburg, Salzburg, Austria

**Keywords:** Normative reasons, Theory of mind, Intentional action, Teleology

## Abstract

There is converging evidence that over the course of the second year children become good at various fairly sophisticated forms of pro-social activities, such as helping, informing and comforting. Not only are toddlers able to do these things, they appear to do them routinely and almost reliably. A striking feature of these interventions, emphasized in the recent literature, is that they show precocious abilities in two different domains: they reflect complex ‘theory of mind’ abilities as well as ‘altruistic motivation’. Our aim in this paper is to present a theoretical hypothesis that bears on both kinds of developments. The suggestion is that children’s ‘instrumental helping’ reflects their budding understanding of practical reasons (in the standard sense of ‘considerations that count in favour of’ someone’s acting in a certain way). We can put the basic idea in the familiar terminology of common coding: toddlers conceive of the goals of others’ actions in the same format as the goals of their own actions: in terms of features of their situation that provide us with reasons to act.

There is converging evidence that over the course of the second year children become good at various fairly sophisticated forms of pro-social activities, such as helping, informing and comforting. Not only are toddlers able to do these things, they appear to do them routinely and almost reliably. As Warneken and Tomasello write, ‘drop an object accidentally on the floor and try to reach for it (..), and infants as young as 14–18 months of age will toddle over, pick it up and return it to you.’ (2009, 397) A striking feature of these interventions, emphasized in the recent literature, is that they show precocious abilities in two different domains: they reflect complex ‘theory of mind’ abilities as well as ‘altruistic motivation’, or so it would seem.

Each of the two developments raises familiar theoretical questions, questions that in the recent literature tend to be treated as separate matters. On the one hand, there are debates about the nature of children’s understanding of goal-directed actions. On the other hand, there is work on how to make sense of young children’s propensity for altruistic interventions, at what is often regarded as a surprisingly young age. Our aim in this paper is to present a theoretical hypothesis that speaks to both kinds of issues. The suggestion is that children’s ‘instrumental helping’ reflects their budding understanding of practical reasons (in the standard sense of ‘considerations that count in favour of’ someone’s acting in a certain way). We can put the basic idea in the familiar terminology of common coding: toddlers conceive of the goals of others’ actions in the same format as the goals of their own actions: in terms of features of their situation that provide us with reasons to act. Making sense of someone else’s intentional activities in these terms is not a detached, theoretical undertaking but has immediate implications for what it makes sense for oneself to do.

One of our tasks will be to fill out the conception of practical reasons needed to make this picture so much as coherent, and to relate the hypothesis to the commonsense psychology of intentional agency. Following recent trends in the philosophy of action and moral psychology, we’ll suggest that, as pre-theoretically conceived, practical reasons combine three salient aspects: they are *normative*, (potentially) *explanatory*, and *public*. A second kind of task will be to examine how our suggestion fares in the light of the experimental evidence, compared to its competitors. The paper will be structured as follows. In section 1 we introduce our suggestion, focusing on the narrow issue of how to characterize young children’s understanding of goals. In section 2 we elaborate on some aspects of the suggestion, in particular the sense in which even young children may think of normative reasons as explanatory. In section 3 we consider the relationship between social cognition and pro-social motivation.

We start with a preliminary remark about helping. Helping *a person* is to be distinguished from helping in a ‘merely causal’ sense, as when you help to wear down the steps by frequently using a staircase. A good example of personal helping is what Svetlova et al. (2010) call ‘empathic helping’, i.e., responding ‘pro-socially to others’ emotional distress’. Svetlova et al. found that children tend to reliably engage in empathic helping somewhat later than in ‘instrumental helping’ (‘assisting another in achieving an action-based goal’). (They tested groups aged 18 and 30 months.) This in turn leads them to ask whether younger children’s instrumental helping was genuinely ‘grounded in other-oriented concern’, or whether ‘they may have simply enjoyed participating in social activities alongside adults, were seeking adults’ approval, or found the activities themselves interesting or fun.’ (Svetlova et al. 2010: 2–3) Note, though, that what Svetlova et al. seem to be suggesting is that there is an interesting development *in children*’*s interpersonal relations* (with the emergence of a more sophisticated engagements with others’ emotional states between 18 and 30 months), not that there is a transition from a stage at which children’s helping is either ‘merely causal’ or in any case quite indifferent to how other agents are faring to a stage at which recognizable interpersonal relations emerge. We’ll come back to the issue of ‘pro-social’ motivation, but for the moment we’ll assume that whatever its motivation, instrumental helping is also a case of personal helping, a (broadly co-operative) response to an agent’s striving to achieve a goal or complete some activity. One basic issue we have to address, then, is how we should characterize children’s grasp of the instrumental structure of the target action.

## 1 Practical reasons

To recognize an action as goal-directed is to find the action intelligible in a distinctive way. One might say: ‘Young children simply register what an agent is trying to achieve; and this they can do without any proficiency in action *explanation*.’ But to recognize someone’s reaching under the table (say) as an attempt to pick up a pen is not just to think of the pen as the end-point of a hand-movement. It is to find the movement intelligible as goal-directed. This doesn’t necessarily imply any great articulacy in answering why-questions. There are arguably more primitive ways in which one might manifest one’s understanding of the goal of an action, to do with expectations and interventions (we’ll come back to this in section 2). Suppose this is right. Then how should we characterize the content of young children’s action understanding? That question raises the larger question of how action explanations in terms of goals actually work. Much depends on what is taken to be the canonical form for stating such explanations. Possible options here include the following:

(1) Max is taking an umbrella in order to stay dry.
(2) Max is taking an umbrella because he wants to stay dry and believes taking an umbrella will enable him to do so.
(3) Max is taking an umbrella because getting wet is unpleasant and using an umbrella is a good way to prevent this.

What (1) brings out nicely is the ‘teleological’ character of the explanation, the key role played by Max’s goal and the instrumental relationship between it and his action. There is, in the developmental literature, a tendency to equate goals with desires. For example, Michael Tomasello and his collaborators propose to use the term ‘goal’ as referring to ‘an internal entity that guides the person’s behaviour (e.g., a mental representation of a desired state such as an open box)’ (Tomasello et al., 2005: 2)^1^ On this view, it is clearly (2) that provides the canonical statement of the target explanation; more specifically: (2) interpreted as invoking causal relations between the agent’s behaviour and her internal states or functional organization.

An alternative analysis of the explanatory force of (1) is suggested by an observation Tomasello et al. make in interpreting a well-known finding reported by Gergely and Csibra. When imitating an adult who is switching on a light by pressing his head against a switch, 18–month-olds will not just switch on the light but will tend to reproduce the bizarre method for doing so used by the adult — unless the adult had his hands occupied (holding a blanket in order to keep warm). As Tomasello et al. remark, ‘apparently, infants assumed that if the adult’s hands were free and he still chose to use his head, then there must be a good reason for this choice (..).’ (2005: 6) The idea here seems to be that children have at least a rudimentary understanding of normative (‘good’) reasons for action, and make sense of goal-directed actions (even unusual ones) in terms of normative reasons (at least they assume the existence of some such reason). This encourages the hypothesis that children understand actions in terms of what Joseph Raz calls the ‘Normative/Explanatory Nexus’. Suppose the facts that p and q give X a good reason to do A, i.e., they are considerations that ‘count in favour of’ X’s doing A, or premises that license the conclusion ‘X should (has reason to) do A’. Then the Normative/Explanatory Nexus holds that the facts that p and q can also be the reason *for which* X is doing A. For ‘every normative reason can feature in the explanation of an action for which it is a reason.’^2^ (Raz 2011: 28)

You might say that normative reasons for action just are (or are provided by) pairs of ‘internal entities’, viz. beliefs and desires. Arguably, though, this is not how commonsense psychology conceives of such reasons. At least in one (common) kind of case, the task for practical reasoning appears to be set by the agent taking some state of affairs or some action to be in some way desirable or valuable or good: for example, fun, interesting, useful, required to keep one’s promise or to do one’s job, decent, fitting etc. etc. . (Anscombe 1957) The task, then, is to find something one can do straight away, doing which will enable one to realize the valuable state of affairs or perform the valuable action. Note, though, that whether the desirability of doing B gives one a reason to do A depends not on one’s *beliefs* about A and B but on the fact of the matter whether doing A will actually result in doing B. Both in relation to ends and to means, a practical reasoner’s attention is usually directed outward. This would suggest a conception of intentional action, not as behaviour caused by pairs of internal entities, but (again in Raz’s words) as ‘a response to the (perceived) normative aspects of the world as they relate to one’ (2001: 153), where the ‘normative aspects’ will, in basic cases, consist of pairs of evaluative features and causal relations. (3) provides an illustrative example.

One interesting implication of this picture is that acquiring a conception of people as intentional agents requires, centrally, becoming able to reason about what people have good reason to do. Another implication, we want to suggest, is that at least an elementary understanding of intentional actions turns out to be available independently of mastery of (2)-type explanations. Young children may find Max’s action intelligible along the lines of (1) and (3), even if they are not yet able to explain actions in terms of beliefs and desires. On this view, the apparently ‘teleological’ character of (1) — the explanatory role accorded to a goal — is not to be reduced to relations of ‘efficient’ causation between mental events and bodily movements. Indeed, at least as understood by young children, (1) is teleological in a sense reminiscent of Aristotle’s conception of ‘teleological causation’. The explanatory force of a goal is inseparable from its evaluative properties: the goal is ‘the good to be achieved’. (Charles 2012: 227)

## 2 The agent’s reason?

Making sense of actions in terms of practical reasons is an example of a ‚personal-level’ explanation. In fact it has often been seen as paradigmatic or even definitive of such explanations. But in the absence of a fully developed ‚theory of mind’, is it still possible to understand the idea of *a person*’*s reason for acting*?

A negative answer to this question may lie behind some recent developmental theories that aim to elucidate children’s grasp of (1) without any appeal to reasons, or persons, at all. One suggestion is that children conceive of the goal of an action as the *function* of a bodily movement. (Butterfill and Apperly 2013) On this view, children’s understanding of (1) can be made perspicuous as follows:

(4) The function of Max’s umbrella-directed bodily movements is to prevent Max getting wet.

‚Function’ is to be understood here on the model of biological functions, as in ‚the function of the heart is to pump blood’. The promise of invoking functions in elucidating children’s conception of goal-directed action is that explanations in terms of functions are, at least on their face, teleological (e.g.,‘The heart is beating *in order to* pump blood’), yet do not invoke an agent’s reasons for acting. That the heart is beating in order to pump blood does not mean that the heart is acting in the light of any consideration counting in favour of beating (say that keeping the blood flowing is a good thing and the best way to achieve this is to beat). Correlatively, we don’t conceive of the heart as an intentional agent, going about its business of making the blood flow properly. Crudely, the function of an organism’s trait is just a matter of the adaptive benefit of the trait to the organism’s ancestors.^3^ The idea, then, is that young children find goal-directed actions intelligible in terms of a kind of ‚non-agentive’ teleology. One sense in which this explanatory schema is ‚non-agentive’ is that the relevant goal is not conceived as *the agent*’*s*, or indeed anyone’s, goal. ^4^ The schema is also ‚non-agentive’ insofar as the explanandum is not actually conceived of as an action, at least in the naive sense of *someone*’*s* doing something (as distinct from a bodily movement).^5^

One problem with this is that it makes the account inhospitable to the idea that children’s early understanding of goal-directed actions plays a role in their developing *interpersonal* relations. Earlier we distinguished personal helping from helping in the ‚merely causal’ sense. It is evidently possible to ‚help’ an object perform its function. For example, one might help the heart to keep the blood circulating by implanting a pacemaker. This would not be a case of helping *someone*, though. An ‚action’ conceived in non-agentive teleological terms is simply not an intelligible target for *personal* helping — helping that is a way to relate to an agent and her purpose.^6^

In the light of this, it seems worth looking more closely into the possibility that the idea of *a person*’*s reason for acting*, or perhaps a simple-minded way to understand the idea, may after all be available even prior to sophisticated ‚theory of mind’ abilities. We can divide this into two sub-questions. (a) If reasons are conceived as ‚normative aspects’ of the world, in what sense are they reasons for particular agents? (b) What might it mean for a reason, thus conceived, to be the reason *for which* an agent is acting in a certain way, or for it to be the agent’s reason for acting in that way?

The right frame of mind for thinking about (a) is surely that there are cases and cases. If you have made a promise to someone this gives *you* a reason to keep the promise. Perhaps it also gives others a reason, at least a reason not to prevent you from redeeming your promise, but intuitively the reason speaks primarily to you. Again, on one (albeit contested) view, what someone has normative reason to do can depend on the intentions they have formed or the projects they have adopted. (See Raz 2011, ch. 8 for discussion.) Of course while these kinds of reasons look ‘agent-specific’ in a more or less obvious sense, they may well be examples of reasons that are only intelligible to mature commonsense psychologists. But consider a more simple-minded kind of reason, say the reason provided by the unpleasantness of getting wet. Note, first, that in this kind of case the requisite psychological competence looks fairly minimal. Simple evaluative concepts such as ‘x is unpleasant’ or ‘doing y is fun’ presumably require some idea of the affective experience involved in undergoing pleasure or discomfort. But this, there is reason to assume, does not outstrip young children’s cognitive skills. There is in fact evidence that 18 month-olds would not only be able to understand that getting wet can be unpleasant but also that what is good (e.g., pleasant) for you depends on who you are. They may be expected to appreciate, for example, that if Max is a frog then getting wet will in fact be pleasant for Max. (Repacholi & Gopnik 1997) Children seem to have some understanding of personal traits such as partialities and dislikes, and of personal needs that arise from hunger, discomfort, and so on. Note, though, that ‘good for Max’ does not mean the same as ‘good, *according to Max*’. Suppose Max is not a frog but Millie is. That getting wet is disagreeable for Max but pleasant for Millie is not to be understood as involving any kind of disagreement. This means it would be a mistake to assume that the fact that some result R (e.g., A not getting wet) is good for A provides an agent-specific reason, a reason only *for A* to bring about R. For we not only value states of affairs or activities but also people. And it is integral to valuing some person A to be disposed to move from ‘R is good for A’ to ‘R is good (simpliciter)’. So the fact that it would be unpleasant for Max to get wet can give *you* a normative reason: it may count in favour, for example, of your telling him that it’s raining (if you know and he doesn’t), or lending him your umbrella (if you have one and he doesn’t), and so on. Thus, what makes reasons agent-specific, at this basic level, is not that an outcome would be good for a particular agent. It is, rather, that the practical implications of the goodness of some outcome for any individual agent will depend on the agent’s practical abilities and opportunities for action. This is one sense in which reasons provided by the ‘normative aspects of the world’ — the shared, or objective, or at least intersubjective world —are reasons for individual agents.

A more serious challenge arises from (b). Intuitively, facts that give someone a normative reason for action can only be expected to become *the agent*’*s reason for acting* if she is aware of these facts and their normative significance. Making this explicit, we may amend (3) as follows:

(5) Max is taking an umbrella because, *as he realizes*, getting wet would be unpleasant and taking an umbrella is a good way to prevent this from happening.

Realizing that p entails knowing that p. But is it possible to master the concept of propositional knowledge without the concept of *belief* (which young children have well-documented trouble with)? We can distinguish four strategies for overcoming this difficulty.

First, one might argue that evidence from ‘indirect’ tests shows even young children have the concept of belief (it’s just that ‘direct’ tests somehow throw them off balance). We’ll set this response to one side. The problem with it, briefly, lies in its assumption that ‘indirect’ measures provide evidence that children are able to have *beliefs* about beliefs, or (equivalently) possess the concept of belief.^7^

Second, one might insist that the concept of knowledge is available independently of the concept of belief, and that there is evidence to suggest that young children grasp the former before the latter.^8^ This is an interesting avenue to pursue, but there are some grounds for scepticism. For one thing, even if knowledge is conceived as a mental state in its own right, rather than as a species of belief (Williamson 2000), there may be reason to think that the two concepts are interdependent. For example, a proper understanding of what it means to know something, it is natural to think, requires understanding what it is to assert something (viz. to make a *claim* to knowledge). But in any case, experimental findings do not lend unequivocal support to the ‘knowledge first’ hypothesis. The evidence regarding young children’s thinking about knowledge presents a very mixed and somewhat confusing picture. (For an illuminating review, see Robinson 2011. For further discussion of the ‘knowledge first’ hypothesis, see Roessler 2013.)

A third option would be to anchor children’s understanding of *an agent*’*s reason for acting* in their conception of desires (often said to emerge early). This suggestion is less straightforward than it may initially seem, though. On one construal, it is circular. Talk of what people want is often just talk of the goals people are pursuing. ^9^ On this construal, that Max wants to stay dry is something that is implied by the fact that he is taking an umbrella in order to stay dry. But it sheds no light on our question, of how children (in the absence of a properly developed propositional attitude psychology) understand the sense in which the considerations that recommend taking an umbrella are *available* to Max, so that he can be expected to act for the reason they give him. Philosophers sometimes use the term ‘desire’ as equivalent to ‘pro-attitude’ in Davidson’s sense. One example of a ‘pro-attitude’ is taking something to be valuable or desirable in some way. Now if children can think of agents as having desires in *that* sense — as taking something to be desirable — this would enable them to make at least partial sense of agents’ awareness or appreciation of the normative reasons they have. The trouble is that there are experimental grounds for skepticism about this analysis. Understanding the concept ‘x takes it to be desirable to do A’ requires a recognition that the construction is neutral on whether x’s doing A *is* in fact desirable. (‘Taking something to be so’ is a non-factive notion.) And there is evidence that young children struggle with this; specifically, that they find it hard to make rational sense of an intentional action if *they* don’t regard achievement of the action’s goal as desirable. (Priewasser, Roessler and Perner 2013)^10^

The fourth option — the one we take to be most promising — develops from an observation by Alison Gopnik and Andy Meltzoff. One-year-olds’ interventions on their environment reflect familiarity with two types of causal relations: physical and psychological. Specifically, young children recognize that whereas mechanical interactions tend to require spatial contact,‘ “action at a distance” is the rule rather than the exception in psychological causality. We influence others by talking, pointing, gesturing and generally communicating.’ (Gopnik & Meltzoff 1997: 141) We’d like to add to this a further observation, and a suggestion. The observation is that when we influence others by communicating with them their resulting actions are frequently intelligible to us in terms of normative reasons. Here are two simple examples. If you draw my attention to an amazing object, my paying sustained attention to the thing will be unsurprising given its look or qualities. If you draw my attention to something you need, again my handing you the object or in some other way ministering to your needs will make sense to you in terms of your need. Our suggestion is that in participating in these sorts of interactions young children may come to think of others as being *responsive to reasons*, even if they initially only have the most minimal grasp of the psychological states *in virtue of which* people are responsive to reasons. One factor children do seem to recognize early on is the direction of others’ attention: they appreciate that the way to get someone to engage with the reason-giving aspects of some object is to draw their attention to the object. Drawing attention to an interesting book can be a way of getting someone to look at the book with me. If I succeed, her activity may be intelligible in terms of, on the one hand, the nature of the book (it’s a nice book to look at) and the direction of her attention (enabling her to engage with the book). The most obvious limitation of this schema is that it only applies to actions that are responses to ‘normative aspects’ of the shared world.^11^ Even so, the schema is not incoherent, and while it hardly amounts to an adequate understanding of what it is to act intentionally, some central ingredients of such understanding are in place: the dependence of normative reasons for action on the agent’s individual practical abilities, a range of evaluative notions, including some understanding of a person’s needs, and the idea that, provided certain enabling conditions are met, people often do what they have reason to do.

## 3 Pro-social cognition

There are several ways in which our hypothesis, that young children understand goaldirected actions in terms of normative reasons, bears on the explanation of earlyprosocial behaviour such as instrumental helping. One, as indicated in the previous section, is that actions, conceived in this way, are at least intelligible targets for ‘personal helping’: they are seen as open to a distinctively ‘personal-level’ type of explanation, in which normative relations, determining what someone has reason to do, play an essential part. Furthermore, there is a close resemblance, on our hypothesis, between the reasoning involved in understanding others’ actions and the reasoning involved in deciding what to do. Only the latter is ‘practical reasoning’ in the sense of reasoning ‘toward action’ (issuing in the formation of an intention). But in a broader sense, even the former may be labeled ‘practical’: it is reasoning about what a particular agent ought to (has reason to) do, given, on the one hand, the desirability of some action or outcome and, on the other hand, the agent’s individual practical abilities and opportunities. In this sense, the two kinds of reasoning use a common format. For example, recall the imitation paradigm discussed in section 1: the adult is switching on a light by bending down and pressing the switch with her head. Children’s practical reasoning about how to switch on the light seems to be informed by their reasoning about how to understand the point of the other’s using her head. On our account, both kinds of reasoning have the same subject matter (the only difference being the identity of the agent whose reasons are in question). Crucially, the result of one kind of reasoning has immediate implications for the other. Whether I have a good reason to use my head depends on the reason you have for using yours. If your reason is that your hands are occupied holding a blanket, it won’t ‘transfer’ to me, since my hands are free. If your hands are free, it is natural to assume that you must be using your head because this is the proper way to operate the device. (The assumption is natural even if I don’t understand the reason why this strange technique is required.) In that case, the same ‘normative aspects of the world’ — making the device blink is exciting (or at least mildly interesting), and the way to do so it to press the switch with one’s head — explain your action and provide me with a reason for mine.

Instrumental helping, from this perspective, is just another example of deriving implications for one’s own actions from the reasons that make others’ actions intelligible.^12^ If you take someone to be acting for the reason that a certain outcome would be desirable, this can affect your own reasons in two ways. It may give you a reason for individual action, if you have the right skills or opportunities to do things (just by yourself) that will promote the outcome. It may also give you a reason to facilitate the others’ activity. For the desirability of the outcome in turn makes it desirable that she should succeed in what she is doing to achieve that outcome. If she encounters an obstacle that you can help her overcome, this gives you a reason to do so. This will not just be a reason to help achieve the desirable outcome (which might be a case of helping in the ‘purely causal sense’). Rather it is, specifically, a reason to help *her* get on with what she is doing. Put differently, her obstacle is perceived as preventing her from getting something she needs, and it would be good to enable her to get. This would suggest that there is a genuine sense in which instrumental helping is, in Svetlana et al’s phrase, ‘grounded in other-oriented concern’. Specifically: in children’s concern with others’ practical needs (which may be less demanding, both to understand and to attend to, than their emotional needs).

On this analysis, the connection between social cognition and ‘pro-social motivation’ is closer than is usually assumed. The two things are often taken to be two independent developmental prerequisites for the emergence of instrumental helping. The prevalence of this ‘dual factor’ approach reflects a widespread conception of commonsense psychology as a theory that provides for explanations of observed behaviour in terms of the agent’s internal functional organization. Social cognition, conceived in these terms, respects the Humean separation between cognition and motivation: the nature of our understanding of goal-directed action is neutral on how that understanding is put to use, whether, for example, in manipulating and exploiting others or in co-operating with them. ^13^ In contrast, our ‘teleological’ account is congenial to Jane Heal’s suggestion that mastery of ordinary psychological concepts such as intentional action, belief or perception is to be understood in terms of the ‘central role’ such concepts play ‘in enabling us to live a shared and (at least partly) cooperative life.’ (2005: 41; see also 2013) Social cognition and a basic disposition for pro-social motivation, on this view, are mutually dependent.

The following evolutionary hypothesis helps to make the contrast between these views of social cognition vivid. Insofar as the adaptive benefit of social cognition lies in the ‘Machiavellian fitness’ individual agents derive from it (Byrne & Whiten, 1988), a theory of mind would be more advantageous than a teleological understanding in terms of normative reasons. It would provide an agent with the means to predict and manipulate others’ behaviour, without being diverted by reasons to help and co-operate. In that sense, theory of mind would outstrip teleology as a tool for cold-minded social intelligence (which should make it the social cognition of choice for psychopaths). In contrast, teleology is intrinsically linked to cooperation, which would confer on it a different kind of adaptive benefit, to do with collaborative action and social cohesion.^14^

We conclude with two caveats. One is that having a disposition for cooperation is one thing, exercising it another. If I make sense of your actions as a response to the ‘normative aspects’ of our shared world, doing so will often draw my attention to facts that give me, not just you, practical reasons. But whether I will act on these reasons is a further question. I may be distracted, or attracted, by other things, and indeed there may be other things I have more pressing reasons to do. While our hypothesis would make the co-emergence of action understanding and a disposition for pro-social motivation unsurprising, it allows that whether children exercise that disposition will depend on a variety of factors. (See Dunfield & Kuhlmeier 2010 for evidence regarding the ‘selectivity” of helping in infancy.)

The other caveat is that as far as *moral* development is concerned, a disposition for cooperation is at best a starting point. Bernard Williams makes this very point in considering an agent who has ‘the notion of doing something *for* somebody’, and sometimes puts that notion into effect. When he does, his motivation is not centred on the satisfaction of his own desires or preferences but on others’ needs: ‘what he wants to do is *to help them in their need*, and the thought he has when he likes someone, and acts in this way, is ‘they need help’, not the thought ‘I like them and they need help’.’ According to Williams, this sort of agent enables us to ‘glimpse what morality needs in order to get off the ground.’ (Williams 1972: 10–11) It is significant, however, that Williams’s characterization is intended to apply to that stock character in moral philosophy, the amoralist. His point is that being inclined to help (significant) others in their need does not necessarily mean morality has already got off the ground. Now a teleologist’s disposition for cooperation is perhaps more systematic and more reliable than the amoralist’s, even on Williams’s sympathetic description of the latter. Nevertheless, while teleology ensures that understanding intentional agents and being disposed to cooperate with them go hand in hand, one crucial question, as far as morality is concerned, is how widely the teleologist will apply that mode of understanding. If morality is to get off the ground, it is not enough to be motivated to cooperate with one’s friends and members of one’s own tribe; one needs to be disposed to treat people in general as having value in themselves. (See Raz 1997 for discussion of this formulation.) And here a simple-minded teleological understanding can actually be something of a hindrance. Those whose actions are not intelligible as a response to the normative aspects of *our* world will not only be seen as unsuitable partners for cooperation but also as opaque, which in turn may prove to be an obstacle to extending natural empathic responses to them. In this way, teleology may foster not only cooperation but also, simultaneously, marginalization. Borrowing a formulation from Williams’s discussion of the amoralist, we might say that to equip a teleologist with ‘some hold on moral considerations’ we need to ‘extend his sympathies’. (1972: 11–12)

## Footnotes

1 Similarly Gopnik and Meltzoff treat as equivalent ‚the same underlying mental state’ and ‚the same goal (1997: 151).

2 For the idea that normative reasons are ordinarily thought of as (potentially) explanatory, and for discussion of how this relates to ‚rationalizing’ explanations in terms of beliefs and desires, see also Hornsby 2008, McDowell 2012, Roessler 2014.

3 ‘On the simplest teleological construal of function, for an action to have the function of bringing about g would be for actions of this type to have brought about g in the past and for this action to occur in part because of this fact.’ (Butterfill and Apperly 2013: 613)

4 Another version of a ‘non-agentive’ teleological account has been advanced in a series of articles by György Gergely and Gergely Csibra. (See e.g., 1998, 2003) The suggestion here is that infants find rational actions intelligible by employing a ‘teleological’ schema of action explanation, central to which is the notion of a ‘well-formed teleological interpretation’. This is said to consist of a relation between a possible future state, situational constraints and the behaviour that is to be explained. It is not clear to us whether the schema is supposed to invoke the biological notion of a function (as in Butterfill and Apperly’s version), and, if not, how to make sense of the explanatory force of appeal to possible future states. In any case, it seems clear that the schema involves no reference to the *agents*’ purpose.

5 Butterfill and Apperly distinguish their ‚more basic notion of goal-directed action’ from the notion of ‚intentional action’, which would require some kind of reason-giving explanation.

6 Another question is whether the notion of behaviour intelligible in terms of its function is actually less demanding than the notion of intentional action. Butterfill and Apperly emphasize that children are not expected to understand ‘the theoretical account of functions, only that [they] can distinguish between things which have different functions in this theoretical sense of ‘function’. (Butterfill and Apperly 2013: 613–4, n. 9) Of course there are numerous ways in which one might distinguish things that as a matter of fact have a different function. (Perhaps they also have different colours.) A natural reading of the claim is that young children are able to distinguish things *by* their functions, or to distinguish the functions of things. While this would of course not require the ability to produce a theoretical account of the nature of functions, it would involve applying and so presumably to some extent understanding the notion of a function. Note that the relevant (biological) notion of a function is not the same as the notion of the intended design of an artefact. The kind of ‘purpose’ involved in the former is neither an agent’s own purpose, nor even a purpose assigned to an object by some other agent, as in the case of artefacts. While there is much work on children’s developing understanding of artefacts, this does not address the question whether young children understand the *biological* notion of a function. Now it might be said that Butterfill and Apperly’s ‘minimal theory of mind’ should not be read as an account of infant’s personal-level action understanding, but as a theory of ‘sub-personal’ or implicit cognition responsible for children’s performance on certain indirect tasks. However, on that construal, the theory would shed no light on the way in which *infants* recognize actions as goal-directed, in the sense of having beliefs and other attitudes about the things people do.

7 For detailed discussion of this response, see Perner and Roessler 2012, Roessler and Perner 2013.

8 See Nagel 2013 for discussion of the philosophical significance of the suggestion that children understand knowledge before understanding belief.

9 See Thompson 2008: 104

10 Another possible construal of the third option would be the suggestion that desires can *be* normative reasons. More precisely: that one wants to do B, together with the fact that doing A will enable one to do B, constitutes a reason for one to do A. This would not, however, solve or even address the current difficulty. For to be the reason for which one is doing A one would still have to *know that one wants to do B*. The difficulty presented by children’s inadequate grasp of propositional attitude psychology remains. It is in any case questionable, as critics of the ‘Humean theory of motivation’ point out, whether commonsense psychology invests the mere psychological fact of someone’s wanting to do something with any normative significance, independently of whatever normative reasons they may have for wanting to do it (reasons that would equally constitute reasons *for action*). As Raz points out, that one wants to murder someone, we would normally assume, it not to be seen as giving one a reason to murder one’s neighbour. If anything, it would give one a reason to see a psychiatrist in order to rid oneself of one’s murderous intentions. (Raz 2011: 159) For critical discussion of the ‚Humean theory’, see also Buss 1999, Schueler 2003, 2009, Stroud 2011, Bridges 2011, Scanlon 2014. Note that arguably the historical Hume’s theory of action was intended neither to provide an account of acting-for-a-reason nor to articulate (as opposed to: correct) the commonsense view of intentional action. (For illuminating discussion of Hume’s theory, see Millgram 1995)

11 For discussion of the evidence for taking young children’s action understanding to be limited in just this way, see Priewasser et al. 2013.

12 Yet another example is the reason not to interfere with your activity. For illuminating discussion of the ‚publicity’ of reasons (the idea that ‚the normative significance of reasons extends across the distinction between agents’ (476)) and its relation to reasons for non-interference, see Wallace 2009.

13 Note that the same seems to be true on a Davidsonian conception of intentional actions as events that are open to a special kind of ‘rationalizing’ explanation. For the ‘reasons’ invoked in such explanations are conceived in such a way that only one agent may coherently be thought to act for them: they are conceived as pairs of mental states, capable of rationalizing only the behaviour of the person whose states they are.

14 Of course once a teleologist takes on board the dependence of someone’s intentional actions on their *perspective* on their reasons — thus can make sense of intentional actions in terms of non-factive attitudes — he will be able to acquire his fair share of Machiavellian intelligence. Then does our evolutionary hypothesis speak to mature commonsense psychology (as distinct from young children’s teleological action understanding)? The answer depends on the account we should give of how the two kinds of understanding are related. Elsewhere we have argued that it would be a mistake to think of them as involving two completely different, unrelated explanatory schemata: rather, mature commonsense psychology involves teleology *plus* an appreciation of the explanatory role of agents’ (limited and possibly ill-informed) perspective on what they have normative reason to do. Crucially, the explanatory conection between non-factive attitudes and intentional actions is not intelligible in isolation; understanding it requires seeing agents as having the ability to be responsive to normative reasons (recognized as such). (Roessler & Perner 2013; Roessler 2014) This would suggest that while coming to appreciate the explanatory role of the agent’s perspective weakens the link between action understanding and cooperation, it does not completely sever it.

## References

[R1] Anscombe GE (1957). Intention.

[R2] Bridges J, Bridges J (2011). Dispositions and rational explanation. The possibility of philosophical understanding.

[R3] Buss S (1999). What practical reasoning must Be if We Act for Our Own reasons. Australasian Journal of Philosophy.

[R4] Butterfill S, Apperly I (2013). How to construct a minimal theory of mind. Mind and Language.

[R5] Byrne RW, Whiten A (1988). Machiavellian intelligence: Social expertise and the evolution of intellect in monkeys, apes and humans.

[R6] Charles D, Shields C (2012). Teleological causation’. The Oxford Handbook of Aristotle.

[R7] Dunfield K, Kuhlmeier V (2010). ‘Intention-mediated selective helping in infancy’. Psychological Science.

[R8] Gergely G, Csibra G (1998). The teleological origins of mentalistic action explanations: a developmental hypothesis. Developmental Science.

[R9] Gergely G, Csibra G (2003). Teleological reasoning in infancy: the naïve theory of rational action. Trends in Cognitive Sciences.

[R10] Gopnik A, Meltzoff AN (1997). Word, thoughts, and theories.

[R11] Hornsby J, Haddock A, Macpherson F (2008). A disjunctive conception of acting for reasons. Disjunctivism.

[R12] McDowell J, Bakhurst D, Hooker B, Olivia Little M (2012). Acting in the light of a fact’. Thinking about reasons.

[R13] Millgram E (1995). Was Hume a Humean?. Hume Studies.

[R14] Nagel J (2013). Knowledge as a mental state. Oxford Studies in Epistemology.

[R15] Perner J, Roessler J (2012). From Infants’ to children’s appreciation of belief. Trends in Cognitive Sciences.

[R16] Priewasser B, Roessler J, Perner J (2013). Competition as rational action: why young children can’t appreciate competitive games. Journal of Experimental Child Psychology.

[R17] Raz J (1997). The Amoralist. Engaging Reason.

[R18] Raz J (2011). From normativity to responsibility.

[R19] Repacholi BM, Gopnik A (1997). Early reasoning about desires: evidence from 14- and 18-month-olds. Developmental Psychology.

[R20] Robinson E, Roessler J, Eilan N, Lerman H (2011). Development of understanding of the causal connection between perceptual access and knowledge State. Perception, causation, and objectivity.

[R21] Roessler J (2013). Knowledge, causal explanation and teleology. Oxford Studies in Epistemology.

[R22] Roessler J (2014). Reason Explanation and the Second-Person Perspective. Philosophical Explorations.

[R23] Roessler J, Perner J, Baron-Cohen S, Tager-Flusberg H, Lombardo M (2013). Teleology: Belief as Perspective. Understanding other minds.

[R24] Scanlon T (2014). Being realistic about reasons.

[R25] Schueler GF (2003). Reasons and purposes.

[R26] Schueler GF (2009). The Humean theory of motivation rejected. Philosophy and Phenomenological Research.

[R27] Stroud B (2011). Engagement and metaphysical dissatisfaction.

[R28] Svetlova M, Nichols s, Brownell CA (2010). Toddlers’ pro-social behavior from instrumental to emphatic to altruistic helping. Child Development.

[R29] Thompson M (2008). Life and action.

[R30] Tomasello M (2005). Understanding and sharing intentions: the origins of cultural cognition. Behavioral and Brain Sciences.

[R31] Wallace J (2009). The publicity of reasons. Philosophical Perspectives.

[R32] Warneken F, Tomasello M (2009). Varieties of altruism in children and chimpanzees. Trends in Cognitive Sciences.

[R33] Williams B (1972). Morality.

[R34] Williamson (2000). Knowledge and its limits.

